# Homophobia and mental health: a scourge of modern era

**DOI:** 10.1017/S2045796021000391

**Published:** 2021-06-29

**Authors:** Antonio Ventriglio, João Mauricio Castaldelli-Maia, Julio Torales, Domenico De Berardis, Dinesh Bhugra

**Affiliations:** 1 Department of Clinical and Experimental Medicine, University of Foggia, Foggia, Italy; 2 Department of Neuroscience, Medical School, Fundação do ABC, Santo André, SP, Brazil; 3 Department of Psychiatry, Medical School, University of São Paulo, São Paulo, SP, Brazil; 4 Department of Psychiatry, School of Medical Sciences, National University of Asunción, Asunción, Paraguay; 5 Department of Mental Health, ASL Teramo, Teramo, Italy; 6 Institute of Psychiatry, King's College, London, UK

**Keywords:** Homophobia, LGBTQI, mental health, minority-stress

## Abstract

Homophobia is still a scourge in the modern era. Despite a greater acceptance of sexual variations and same-sex marriage in many countries, homophobia is widely sustained by religious, political and cultural values and beliefs at individual and social level. Most of homophobic attitudes are based on the principle of heteronormativity according to which heterosexuality is the standard for legitimating social and sexual relationships and homosexuality is considered as an abnormal variant. Homophobia may be also recognised at institutional level (state-sponsored homophobia, social homophobia) and supported by laws or religious beliefs. Moreover, internalised homophobia (IH) is defined as the inward direction of societal homophobic behaviours at individual level and refers to the subjective psychological impact of these negative attitudes. In fact, IH is significantly associated with a high prevalence of internalising mental disorders such as depression, anxiety, stress/trauma-related disorders, etc. We believe that a set of immediate actions are needed in order to contrast homophobia and its impact on mental health, in particular political initiatives, educational trainings and scientific research should be promoted with a specific focus on mental health needs of people target of homophobia.

## Homophobia

Despite an increasing acceptance of homosexuality and sexual variations in the last decades with greater recognition of same-sex marriage and humans rights, also sustained by LGBTQI (Lesbian, Gay, Bisexual, Transgender, Queer, Intersexual) activism and socio-political changes, homophobia is still a scourge in the modern era and the fight against this social and cultural phenomenon ought to be an immediate priority (Poushter and Kent, 2020). In fact, attitudes of acceptance may vary around the globe with a higher level in the American and Western European countries, and lower in Eastern Europe, the Middle East, Russia and part of Africa.

*Homophobia* is defined as a range of behaviours, feelings, negative attitudes towards sexual variations and people identified or perceived as LGBTQI (Renzetti and Edleson, 2008). The origin of the term is traced back to Weinberg, a Jewish-American psychologist of the 1960s (Grimes, 2017), and refers to a composite term deriving from the words *homo-sexuality* and *phobia*, which is a Greek term (φόβος) meaning ‘fear’ or ‘aversion’ or ‘dread’. This term has appeared in the printed media in the following years: *homophobic panic* on *Time* (US) magazine in 1969, *homophobia* on *The Times* (London, UK) in 1981 until it became of common use globally (Longley, 1981). In 1972, Weinberg published his book entitled *Society and the Healthy Homosexual* with a broader analysis on homophobia and its consequences on health. It is of interest that one year later, in 1973, the category homosexuality has been removed from the upcoming version of Diagnostic and Statistical Manual of Mental Disorders (DSM-III edition; 1980): the task force coordinated by Professor Robert Spitzer reformulated the concept of homosexuality and sexual orientation disturbance proposing the differentiation between the normal sexual variant from other same-sex attractions characterised by experienced distress or psychological disturbance.

Some other authors have also disputed that homophobia is not consistent with the definition of *phobia* that should be intended as an ‘intense, somewhat illogical, or abnormal fear of a specific thing or situation’: homophobia is sustained by negative attitudes, emotions as well as religious, political and cultural values and beliefs (Plummer, 2016). In fact, many factors may lead to homophobia: prejudice, ignorance, fear, hate, mistrust, discomfort. Undoubtedly, homophobia relies on religious, political and cultural values and beliefs, as well as, generally, negative attitudes towards homosexuality are inspired by the concept of heteronormativity. *Heteronormativity* is a principle according to which heterosexuality is the standard for legitimating social and sexual relationships whereas homosexuality is a variant and may be seen as an abnormality (Berlant and Warner, 1998). Homophobia may be named as *lesbophobia* (directed against lesbians), *biphobia* (against bisexuals), *transophobia* (against transsexuals). Intolerance against any diversity (sexual variation, race, political and religious minorities, etc.) may be individual or affecting the community or institutions. It has been proposed that at an individual level, homophobia as well as other aversions against minority may be linked to a psychopathological construct such as the *intolerant personality disorder* (Guindon *et al*., 2003) with the following characteristics: (a) a rigid set of beliefs and values based on the superiority of race or religion, culture, sexual orientation, etc.; (b) lack of empathy; (c) antagonism and hostility against a specific target population; (d) aversion and efforts to block, contrast, impede people considered to be inferior; (e) use of power to contrast the intolerable people or ideas; (f) a sense of entitlement based on the sense of being part of a superior group; (g) disregard for human rights; (h) lack of remorse.

At societal level, many categories and definitions of homophobia have been proposed as: *institutionalised* homophobia, *state-sponsored* homophobia, *social* homophobia and *internalised* homophobia (IH) (Frost and Meyer, 2009).

*Institutionalised homophobia:* institutions may be strongly based or oriented on specific cultural, religious as well as prevalent ideologies. All these factors may consequently affect social attitudes towards minorities or part of population. Religions in the world may show a variable approach to the theme of sexual variations with a range of degrees of acceptance. Catholic religion has modulated teachings and positions towards homosexuality over time. An implicit condemn of same-sex attractions might be found in the Old and New Testaments of The Bible (e.g. Leviticus 18:22; the story of the judgment of Sodom and Gomorrah; King, 1976); the Catechism of Catholic Church also disapproved homosexuality and same-sex acts stating they may be considered as contrary the natural law (The Vatican, 2020). Pope Francis, after his election in 2013, opened to homosexuality and stated that Catholic Church should love people regardless of their sexual orientation: in 2019, he specified that homosexual orientations are not considered as a sin by the Catholic teaching even if homosexual acts are a sin; later in 2020, in a documentary named *Francesco*, he stated that homosexuals have the right to be part of the family and are God's children; he also opened to civil unions among same-sex lovers (San Martín, 2019; Horowitz, 2020).

Homosexuality is forbidden by Islam and is considered a crime under Sharia Law: in Afghanistan, same-sex acts are punished with the death penalty under the Taliban as well as gay people have been persecuted by Islamist forces in Iraq or Syria (Mc Claughlin, 2016). The International Lesbian and Gay Association (ILGA) reported that about 80 countries around the world continue to consider homosexuality as illegal (2009). Penalisation as well as criminalisation of homosexuality in these countries, including also persecution of LGBT, is also considered state-sponsored homophobia (Bruce-Jones and Lucas Paoli, 2011). Many examples of state-sponsored discrimination may be described in the history: the Medieval Inquisition, the Republic of China (under the Qing Dynasty), Soviet Union (under Lenin), Nazi Germany, North Korea, Zimbabwe, etc.

In communities based on homophobia, the fear of being identified as gay is higher and may be recognised as a social-homophobia: this may lead to an exhibition of heterosexual behaviours, congruent with the heteronormative culture, with a distancing from gay people in order to reaffirm a conventional social role and gain social validation (Eguchi, 2006).

*Internalised homophobia (IH):* may be defined as the inward direction of societal homophobic attitudes at individual level. It is a psychological construct including the internalisation of negative attitudes conflicting with the self-regard and leading to a self-denigration or identification with the heterosexual beliefs as theorised by Allport (1954). This process may cause discomfort with the own sexual orientation seen as ego-dystonic: personal desires and attractions are seen odds with the individual self-image and may cause extreme stress, repression and discordance (Newcomb and Mustanski, 2010). As further discussed, IH may lead to repression of the own desires, internalising mental health problems, clinical depression and a higher risk of suicide (Newcomb and Mustanski, 2010).

## Homophobia and mental health

We argue that the impact of homophobia on mental health is understated and undetected in the clinical setting as well as poorly described in the literature.

In 2010, a meta-analytic review of literature conducted on the relationship between IH and mental health among LGBTQIs concluded that IH is significantly associated with internalising mental disorders (e.g. depression, anxiety, stress/trauma-related disorders, obsessive-compulsive disorder, eating and dissociative disorders) (Newcomb and Mustanski, 2010).

Several authors include the homophobic experience into the framework of ‘*minority stress model*’: minority stress derives from the conflict between being a minority and dominant social and cultural values, and may be based on homophobic experiences, harassment, maltreatment, discrimination and victimisation, all affecting individuals' physical and mental health outcomes (Meyer, 1995).

Newcomb and Mustanski (2010) meta-analysed 31 articles on the association between IH and mental health covering a total sample of 5831 LGBTQI individuals and reported that homophobic experiences were associated with high rates of internalising mental disorders, mostly described in older individuals and based on prevalent depressive symptomatology. Interestingly, Van Beusekom *et al*. (2018), after assessing 724 LGB individuals, proposed that homophobic stigmatisation and IH are significant *mediators* of the association between gender non-conformity and the onset of mental health issues: subjects reporting less homophobic experiences have shown lower mental health morbidity. Lorenzi *et al*. (2015), assessing LGB subjects in Belgium and Italy, found that social support is a protective mediator between IH and anxiety as well as depressive symptoms in their path-analysis model. Also, higher levels of IH and social heteronormativity have been associated with a significant increase of sexual risk behaviours and increased incidence of sexually transmitted diseases among LGBs (Perez-Brumer *et al*., 2019).

IH also affects the quality of life and life satisfaction: as reported by Wen and Zheng (2019), there is a significant association between homophobic experiences and lower life satisfaction among LGB individuals (*N* = 528), as well as their mental health status was reported as a statistically significant mediator. These findings confirm what is reported by a recent high-quality review on suicidal behaviours among sexual minorities: authors listed homophobia and micro-aggressions as recognised *specific* risk factors for suicide among LGBTs (Poštuvan *et al*., 2019).

Alongside the impact of homophobia on mental health, it is of note that high homophobic and discriminatory attitudes have been found among health care professionals with potential negative effects on the quality of care and therapeutic relationship towards LGBT patients (Taskiran Eskici *et al*., 2021).

## What's next

We argue that a set of immediate actions are needed in order to fight homophobia and reduce the impact of social pressure on LGBTs' mental health: (a) a synergy between governments, LGBT-rights organisations, mental health associations in order to promote campaigns against homophobia and raise awareness on the impact of discrimination and non-acceptance on mental health; (b) educational trainings on homophobia for secondary schools and universities; (c) specific courses on health and mental health of LGBTQIs and related issues among health care professionals, to be added in their core curriculum; (d) promotion of the detection and measurement of homophobia in the clinical setting (including mental health services); (e) training on specific instruments of measurement (e.g. Short Internalised Homonegativity Scale, Nungesser Homosexual Attitudes Instrument, Internalised Homonegativity Inventory) and development of more specific tools, (f) promotion of more research on LGBTQIs mental health and their health unmet needs.
